# Supplementary material for: Discovery of a non-canonical GRHL1 binding site using deep convolutional and recurrent neural networks
Source: BMC Genomics. 2023 Dec 4;24:736. doi: 10.1186/s12864-023-09830-3 (PMC10696883; doi:10.1186/s12864-023-09830-3)
Supplement: Supplementary file 1 — Supplementary Material 1 [file 12864_2023_9830_MOESM1_ESM.docx]

# **SUPPLEMENTARY DATA**


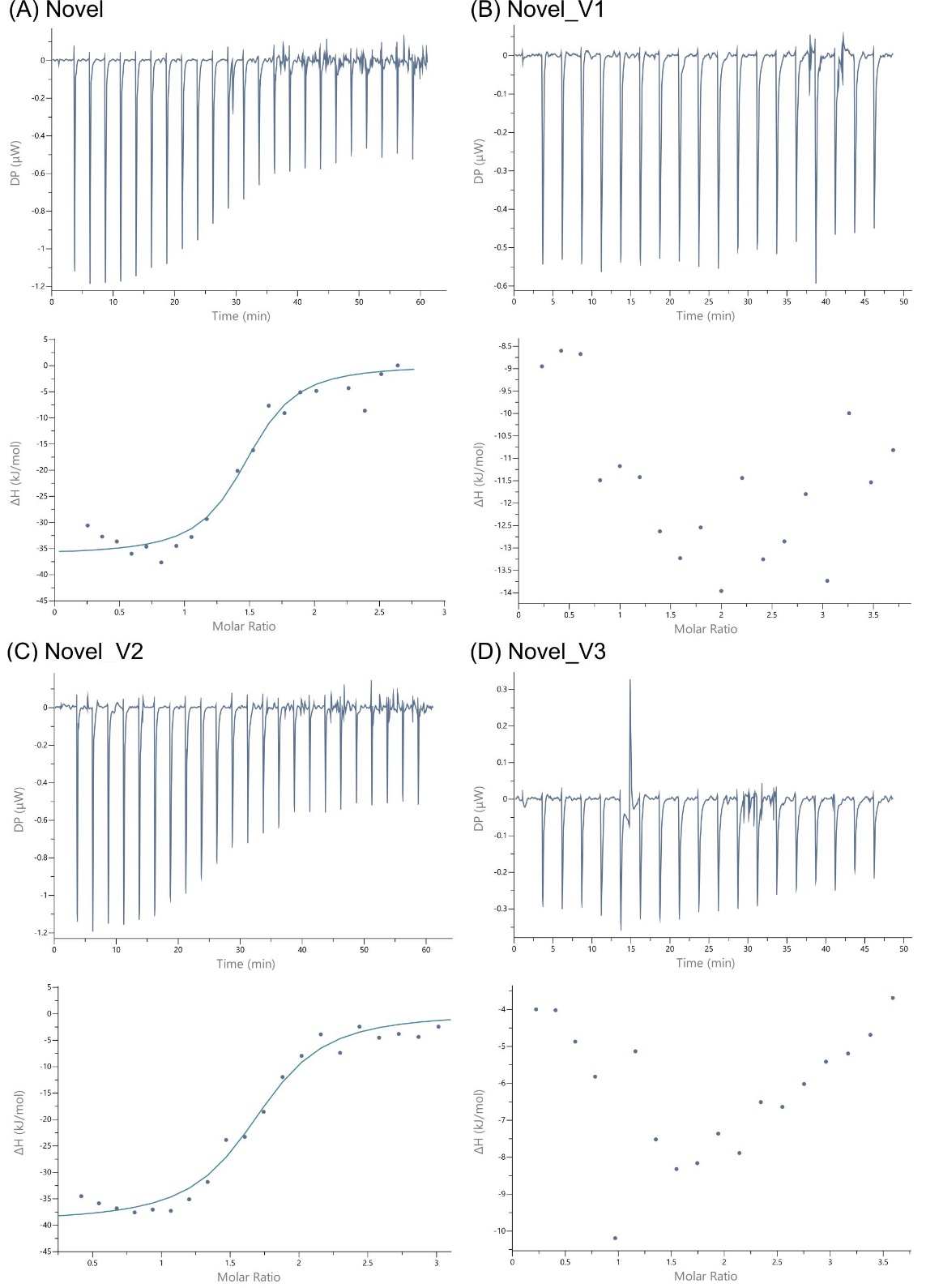


Figure S1: Calorimetric titration of Novel_V1-4 with GRHL1. The top panel shows the raw data obtained from 24 or 19 consecutive 1.5 or 2 μl injections of 165 μM or 200 μM GRHL1 solution into the sample cell containing ~10 μM of dsDNA. The binding isotherm of the bottom panel results from plotting heat peak areas against the dsDNA: protein molar ratio. The blue line represents the best fit of a model of one set of binding sites.


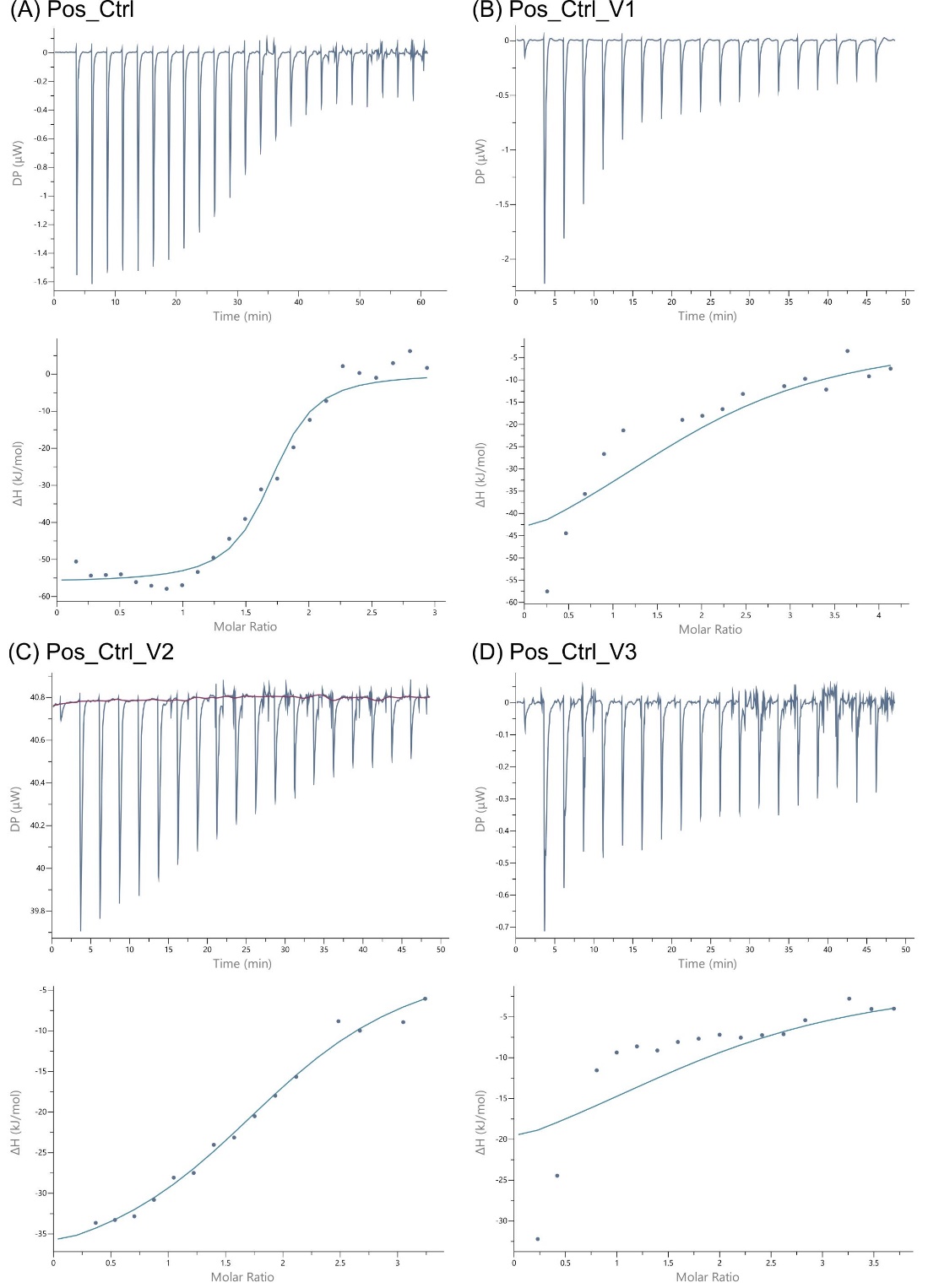


Figure S2: Calorimetric titration of Pos_Ctrl_V1-4 with GRHL1.The top panel shows the raw data obtained from 24 or 19 consecutive 1.5 or 2 μl injections of 165 μM or 200 μM GRHL1 solution into the sample cell containing ~10 μM of dsDNA. The binding isotherm of the bottom panel results from plotting heat peak areas against the dsDNA: protein molar ratio. The blue line represents the best fit of a model of one set of binding sites.
